# Impact of Enhanced External Counter-pulsation Therapy on Glycaemic Control in People With Prediabetes and Type 2 Diabetes Mellitus: A Systematic Review and Meta-analysis

**DOI:** 10.17925/EE.2023.19.2.8

**Published:** 2023-10-02

**Authors:** Lakshmi Nagendra, Deep Dutta, Meha Sharma, Harish Bg

**Affiliations:** 1. Department of Endocrinology, JSS Academy of Higher Education and Research, Mysore, India; 2. Department of Endocrinology, Center for Endocrinology Diabetes Arthritis & Rheumatism (CEDAR) Superspeciality Healthcare, Dwarka, New Delhi, India; 3. Department of Rheumatology, Center for Endocrinology Diabetes Arthritis & Rheumatism (CEDAR) Superspeciality Healthcare, Dwarka, New Delhi, India; 4. Department of Anaesthesiology, JSS Academy of Higher Education and Research, Mysore, India

**Keywords:** Coronary artery disease, external counter-pulsation therapy, glycaemic control, randomized controlled trials, systematic review, type 2 diabetes mellitus

## Abstract

**Background**: Enhanced external counter-pulsation (EECP) therapy is approved for refractory angina in coronary artery disease (CAD). EECP is being explored as a treatment modality in type 2 diabetes mellitus (T2DM). **Methods**: The Embase, Web of Science, Cochrane Library, MEDLINE (PubMed), ClinicaltTrials. gov, CNKI database, Clinical Trials Registry-india (CTRI), and Google Scholar databases were searched for randomized controlled trials (RCTs) involving patients receiving EECP therapy in the intervention arm. The primary outcome was the changes in glycated haemoglobin (HbA1c). The secondary outcomes were the changes in blood glucose parameters, inflammatory markers and any adverse events. **Results**: Data from 3 RCTs involving 71 people with T2DM/prediabetes was analysed to find out the impact of EECP therapy compared with placebo. As compared with placebo, patients receiving EECP had significantly lower HbA1C immediately after completion of therapy (mean difference [MD] -0.70%, 95% confidence interval (CI) -0.95. -0.45;p<0.00001), at 2–4 weeks post completion of therapy (MD -1.04%, 95%CI -1.32. -0.77; p<0.00001) and 7–12 weeks after therapy completion (MD -0.98%, 95% CI -1.22, -0.74; p<0.00001). EECP therapy was well tolerated without any increased side effects (risk ratio 2.36, 95% CI 0.11–52.41; p=0.59. **Conclusion**: EECP therapy is effective in blood glucose and pressure lowering over at least 7–12 weeks of therapy completion. Blood glucose and pressure should be monitored with suitable modulation of drug doses to prevent hypoglycaemia and hypotension in patients with angina undergoing EECP therapy. The PROSPERO registration number is CRD42023434533

## Highlights

- Enhanced external counterpulsation (EECP) is evaluated for glycaemic control in type 2 diabetes.
- Glycated haemoglobin reduction immediately after EECP therapy conclusion, after 2–4 weeks and after 7–12 weeks was -0.70%, 1.04% and 0.98%, respectively.
- EECP is associated with a significant lowering of blood pressure.
- EECP significantly lowers the levels of high-sensitivity C-reactive protein, a measure of systemic inflammation.

Enhanced external counterpulsation (EECP) therapy is a noninvasive, nonpharmacological outpatient/daycare treatment approved by the US Food and Drug Administration therapy that has been used for treating refractory angina in people living with coronary artery disease (CAD) for more than three decades now.^1,2^ During EECP therapy, pneumatic compression cuffs are applied to the calf and the lower and upper thigh of each leg.^2^ These cuffs are inflated sequentially through computergenerated signals while synchronized to the patient's R wave on the electrocardiogram. EECP therapy leads to retrograde blood flow in the aorta, resulting in a diastolic augmentation of blood flow and improved coronary perfusion pressure during diastole.^2^ Thereafter, the cuffs simultaneously deflate before the onset of systole, decreasing vascular resistance, assisting with systolic unloading and decreasing cardiac workload, thereby reducing angina.^2^ Mechanistically, shear stimulus in the femoral and brachial arteries results in increased endothelial nitric oxide (NO) production, decreased systematic inflammation (high-sensitivity C-reactive protein) and improved endothelin-1 levels.^3,4^ The increased production of vasodialators such as NO results in reduced myocardial oxygen demand, increased venous return and cardiac output, improved endothelial function, promoted coronary collateral development and recruitment, and prolonged time to exercise-induced ST depression.^4^ Seven weeks of EECP therapy have been found to reduce angina symptoms and frequency in patients with CAD; however, the benefits of EECP therapy have been found to be more blunted in people with diabetes.^5^

Skeletal muscles are a major organ for glucose disposition and storage following meal-induced hyperglycaemia. Physiologically, glucose is uptaken and stored in skeletal muscles through three major mechanisms: insulin-mediated glucose uptake, which is impaired in people with type 2 diabetes mellitus (T2DM) due to skeletal muscle insulin resistance;^6^ muscle contraction-mediated glucose uptake, which explains the beneficial impact of glycaemic control in T2DM;^7^ and NO-mediated glucose uptake.^8^ NO-mediated glucose uptake may explain the beneficial impact of EECP therapy on glycaemia in T2DM, as EECP therapy works by enhancing NO formation, thus leading to increased NO-mediated glucose uptake in skeletal muscles. Several randomized controlled trials (RCTs) evaluating the effect of EECP therapy on glycemic parameters in patients with T2DM have been published.^9–11^ However, no systematic review and meta-analysis providing a holistic view of the role played by EECP therapy in treating T2DM has been published to date. Hence, we conducted a systematic review and meta-analysis to evaluate the safety and efficacy of EECP therapy for glycaemic control in patients with T2DM compared with control patients.

## Methods

The systematic review and meta-analysis were conducted following the Preferred Reporting Items for Systematic Reviews and Meta-Analyses (PRISMA) guidelines. The study was registered in the International Prospective Register of Systematic Reviews (PROSPERO); the registration number is CRD42023434533. For this systematic review and metaanalysis, we considered RCTs involving people with T2DM or prediabetes receiving EECP therapy in the study group compared with participants receiving placebo or any other medication in the control group. Patients with other forms of diabetes like type-1 diabetes, gestational diabetes, and other rarer monogenic causes of diabetes were excluded. The primary outcome of the meta-analysis was changes in glycated haemoglobin (HbA1c) from baseline. The secondary outcomes were changes in fasting plasma glucose (FPG), post-prandial glucose (PPG), lipid parameters, inflammatory markers and any adverse events. Separate analyses were performed for controls receiving placebo, labelled as placebo or passive control group, and controls receiving other antidiabetes medications, labelled as active control group.

We systematically searched the Embase, Web of Science, Cochrane Library, MEDLINE (PubMed), ClinicalTrials.gov, CNKI, Clinical Trials Registry-I ndia (CTRI) and Google Scholar databases for the following keywords or MeSH terms: (enhanced external counter-pulsation therapy) OR (external counter-pulsation therapy) OR (counter-pulsation therapy) for the articles published untill May 2023. Methodologic details regarding literature review have been elaborated in a previous meta-analysis published by our group.^12^ The risk of bias assessment was done by three authors independently using the risk of bias assessment tool in the Review Manager (RevMan) version 5.4 software. The different types of bias that were assessed had been elaborated in previous metaanalyses conducted by our group: selection bias, performance bias, detection bias, attrition bias, reporting bias and other bias.^12,13^ Other bias included sources of funding, especially when there is a pharmaceutical/ organization involvement in the manufacture and sale of EECP devices, and conflict of interests. A random effects model was used for the metaanalysis. Forest plots were generated to assess the heterogeneity for all outcomes. Specifically, heterogeneity was analysed using the Χ^2^ test on N-1 degrees of freedom, an alpha of 0.05 used for statistical significance and the I^2^ test.^13,14^ The certainty of the evidence of the major outcomes in this meta-analysis was evaluated using the Grades of Recommendation, Assessment, Development and Evaluation (GRADE) approach.^15^ A table highlighting the grading of key outcomes was generated using the GRADE software. The details have been elaborated elsewhere.^13^ Publication bias was assessed for key outcomes using funnel plots (Supplementary Figure S1).^16^

## Results

**Figure 1:**
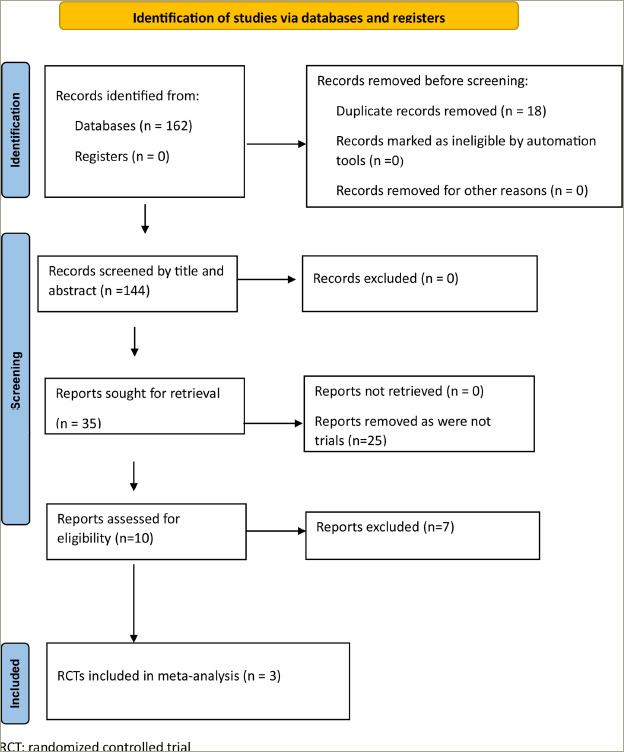
The PRISMA flow diagram for the systematic review

A total of 162 articles were found from databases after the initial search (*Figure 1*). Eighteen duplicates were removed. After screening the titles and abstracts of the remaining 144 articles, the search was reduced to 35 studies, which were evaluated in detail for inclusion in this meta-analysis (*Figure 1*). Finally, three RCTs were found to fulfil all the inclusion and exclusion criteria and were analysed in our systematic review and meta-analysis.^9,10,17^ The three articles included in our systematic review and meta-analysis are described in detail in *Table 1*.

In 2 of the RCTs, the study population was patients with T2DM.^9,10^ In the study by Martin and Braith, 1 of 6 participants (17%) in the control group and 4 of 12 (33%) participants in the EECP therapy group had prediabetes; the remaining participants had T2DM.^17^ All three trials compared EECP therapy with placebo over and above the standard of care in T2DM.^9,10,17^ The study by Hoong et al. did not have a comparator group and hence was excluded from the analysis.^11^

**Figure 2:**
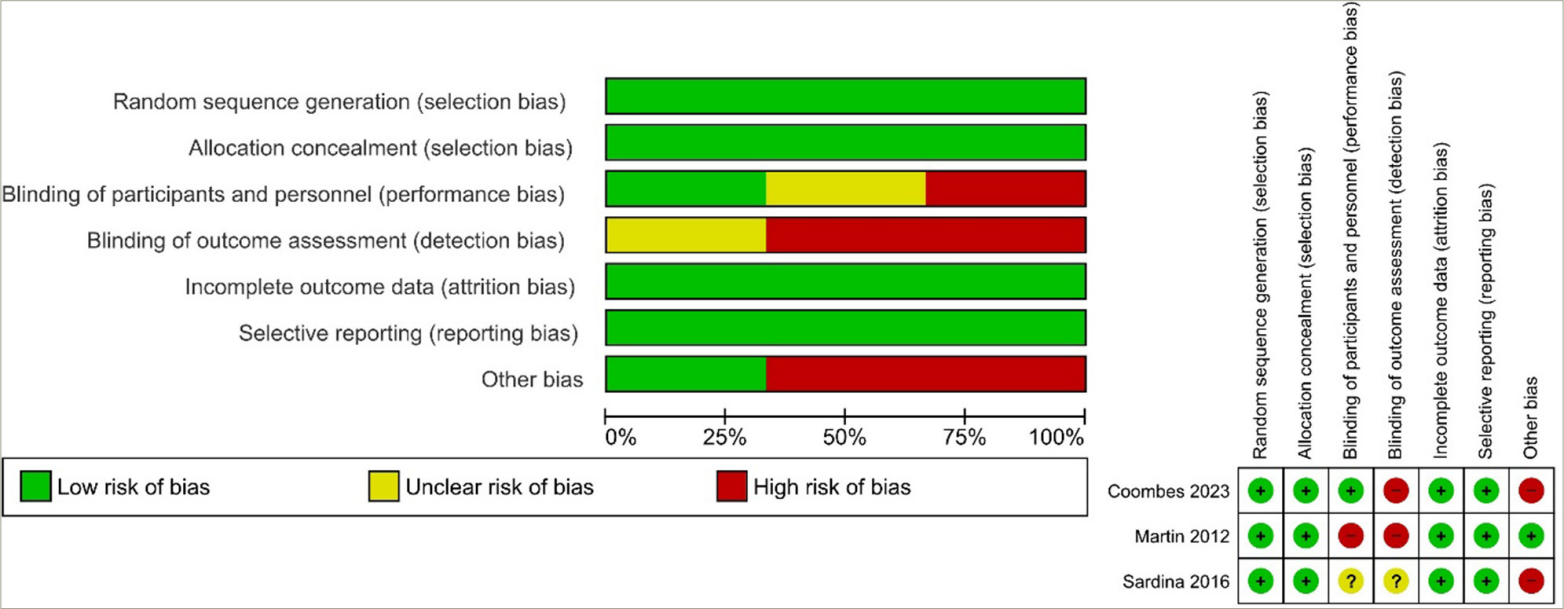
(a) Risk of bias graph: the review authors' judgements about each risk of bias item presented as percentages across all included studies. (b) Risk of bias summary: the review authors' judgements about each risk of bias item for each included study

The risk of bias in these studies is summarized in *Figures 2a and b*. The risk of selection, attrition and reporting bias was judged to be low in all three studies.^9,10,17^ There was a low risk of performance bias in one out of the three studies.^9^ The risk of detection bias was high in two of the three studies,^9,17^ while the risk of bias was unclear in Sardina et al.^10^ Other bias was judged to be at high risk in two out of the three studies.^9,10^

### Outcomes after therapy completion

**Figure 3:**
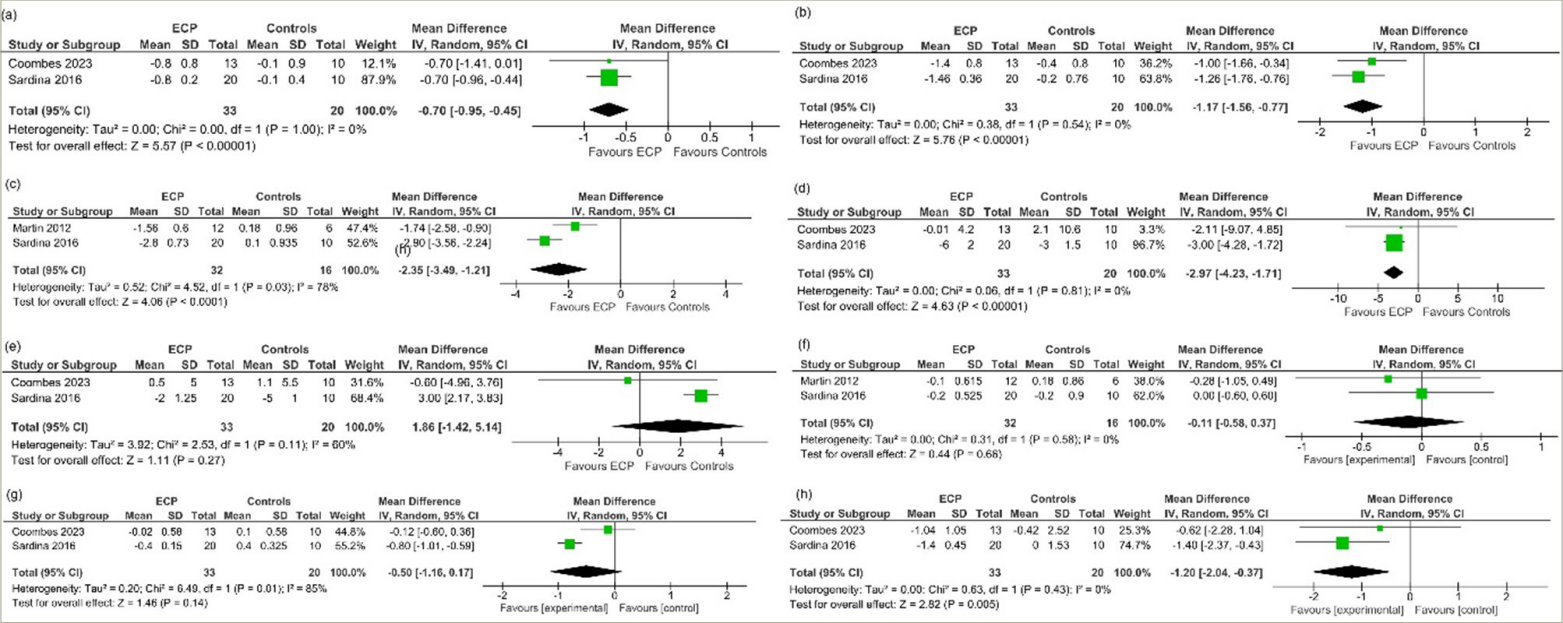
Forest plot highlighting the impact of enhanced external counter-pulsation therapy compared with passive control group (placebo) at completion of therapy on (a) glycated haemoglobin; (b) fasting plasma glucose; (c) 2 hour post-prandial glucose; (d) systolic blood pressure; (e) diastolic blood pressure; (f) body mass index; (g)interleukin-6; (h) high-sensitivity C-reactive protein

Therapy was considered completed at around 35 sessions of EECP therapy over 7–8 weeks. Data from three studies involving a total of 71 people with T2DM or prediabetes undergoing EECP therapy were analysed to determine the impact of EECP therapy compared with the control participants.^9,10,17^ At therapy completion, patients receiving EECP therapy had significantly lower HbA1c levels (mean difference [MD] -0.70%, 95% confidence interval [CI] -0.95, -0.45; p<0.00001; I^2^=0% [low heterogeneity (LH)]; high certainty of evidence [HCE]; *Figure 3a*), FPG (MD -1.17 mmol/l, 95% CI -1.56, -0.77); p<0.00001; I^2^=0% [LH]; HCE; *Figure 3b*) and 2-hour PPG (MD -2.35 mmol/l, 95% CI -3.49, -1.21; p<0.0001; I^2^=78% [moderate heterogeneity (MH)]; moderate certainty of evidence; *Figure 3c*) levels compared with the placebo or passive control group. SBP was significantly lower in the EECP therapy group compared with the placebo or passive control group (MD -2.97 mm Hg, 95% CI -4.23, -1.71); p<0.00001; I^2^=0% [LH]; *Figure 3d*), while DBP was similar between the two groups (MD 1.86 mm Hg, 95% CI -1.42, 5.14; p=0.27; I^2^=60% [MH]; *Figure 3e*). Body mass index (MD -0.11, 95% CI -0.58, 0.37; p=0.66; I^2^=0% [LH]; *Figure 3f*) and interleukin-6 (MD -0.50 pg/ml, 95% CI -1.16, 0.17; p=0.14; I^2^=85% [high heterogeneity (HH)]; low certainty of evidence; *Figure 3g*) were similar between groups. C-reactive protein (MD -1.20; 95% CI -2.04, -0.37; p=0.005; I^2^=0% [LH]; *Figure 3h*) was significantly lower in the EECP therapy group compared with the placebo or passive control group after therapy completion. Adverse effects were similar between the groups (risk ratio 2.36, 95% CI 0.11–52.41; p=0.59; LH; HCE). The only adverse event related to EECP therapy was chafing on the legs where the cuffs had been applied. No patient experienced withdrawal from undergoing EECP therapy at trial completion. In one trial, the participants reported that they had sensitive skin, and wearing cotton pants and applying moisturiser during therapy helped to reduce their symptoms.^9^

### Outcomes at 2–4 weeks after therapy completion

**Figure 4:**
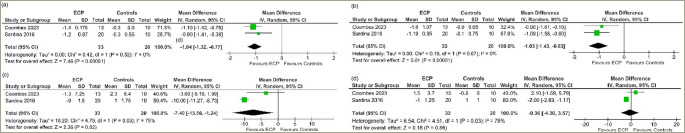
Forest plot highlighting the impact of enhanced external-counterpulsation therapy compared with placebo or passive control group at 2–4 weeks on (a) glycated haemoglobin; (b) fasting plasma glucose; (c) systolic blood pressure; (d) diastolic blood pressure

Data from 2 studies involving 53 people with T2DM were analysed to determine the impact of EECP therapy compared with placebo at 2–4 weeks after therapy completion.^9,10^ Compared with patients in the placebo or passive control group, patients receiving EECP therapy had significantly lower HbA1C (MD -1.04%, 95% CI -1.32, -0.77; p<0.00001; I^2^=0% [LH]; HCE; *Figure 4a*), FPG (MD -1.03 mmol/l, 95% CI -1.43, -0.63; p<0.00001; I^2^=0% [LH]; *Figure 4b*) and SBP (MD -7.40 mm Hg, 95% CI -13.56, -1.24; p=0.02; I^2^=79% [MH]; *Figure 4c*) levels at 2–4 weeks after therapy completion. DBP (MD -0.36 mm Hg, 95% CI -4.30, 3.57; p=0.86; I^2^=78% (MH); *Figure 4d*) was similar between the groups.

### Outcomes at 7–12 weeks after therapy completion

**Figure 5:**
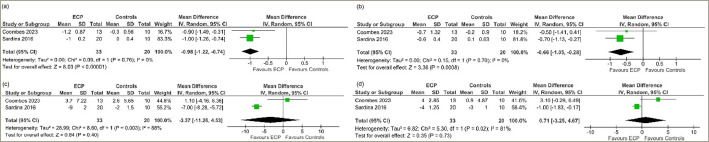
Forest plot highlighting the impact of enhanced external-counterpulsation therapy as compared with placebo or passive control group at completion of therapy at 7–12 weeks on (a) glycated haemoglobin; (b) fasting plasma glucose; (c) systolic blood pressure; (d) diastolic blood pressure

Data from 2 studies involving 53 people with T2DM was analysed to determine the impact of EECP therapy compared with placebo at 7–12 weeks after completion of therapy.^9,10^ Compared with patients in the placebo or passive control group, patients receiving EECP therapy had significantly lower levels of HbA1C (MD -0.98%, 95% CI -1.22, -0.74; p<0.00001; I^2^=0% [LH]; HCE; *Figure 5a*) and FPG (MD -0.66 mmol/l, 95% CI -1.05, -0.28; p=0.0008; I^2^=0% [LH]; *Figure 5b*). SBP (MD -3.37 mm Hg, 95% CI -11.26, 4.53; p=0.40; I^2^=88% [HH]; *Figure 5c*) and DBP (MD 0.71 mm Hg, 95% CI -3.25, 4.67; p=0.73; I^2^=81% [HH]; *Figure 5d*) levels were similar between the two groups.

**Table 1:** Patient characteristics from the three randomized controlled trials retrieved in the systematic review

	Coombes et al.^9^		Sardina et al.^10^		Martin and Braith^17^	
	EECP (n=10)	Placebo (n=13)	EECP (n=20)	Placebo (n=10)	EECP (n=12)	Placebo (n=6)
Age (SD)	61.0 ± 8.9	60.8 ± 8.2	63.0 ± 2.5	56.6 ± 3.0	64.30 ± 11.95	64.00 ± 2.76
Females (%)	0%	60%				
BMI (kg/m^2^)	34.50 ± 7.00	33.2 ± 6.7	32.8 ± 1.0	32.8 ± 1.9	30.33 ± 1.26	29.49 ± 1.78
SBP (mm Hg)	132.4 ± 12.8	128.5 ± 11.3	130.0 ± 4.0	131.0 ± 5.0		
DBP (mm Hg)	77.7 ± 6.4	75.8 ± 10.3	76.0 ± 3.0	82.0 ± 3.0		
HbA1c (%)	8.3 ± 2.1	8.1 ± 1.3	7.3 ± 0.4	7.6 ± 0.8		
FPG (mmol/L)	8.3 ± 2.6	8.0 ± 1.3	10.2 ± 0.79	10.7 ± 1.49		
Exclusion criteria	Unstable angina; recent (within the past 4 weeks) myocardial infarction; coronary artery disease; uncompensated heart failure (NYHA functional classification II–IV); severe valvular illness; pulmonary disease; uncontrolled hypertension (systolic blood pressure >200 mmHg and/or diastolic blood pressure >110 mmHg); renal failure (chronic kidney disease stages IV or V); orthopedic/neurological limitations; cardiomyopathy; weight loss surgery in the previous year; planned operations during the research period; drug or alcohol abuse; current or expected pregnancy		Insulin dependence for glycaemic control; any major illness in the prior 3 months; participation in moderate intensity exercise for 20 min ≥2 times per week; history of deep vein thrombosis, phlebitis, stasis ulcer or pulmonary embolism; aged <18 years old; uncontrolled hypertension or systemic hypotension; cardiac arrhythmia that would interfere with ECG synchronization of EECP therapy; severe heart failure; aortic valve insufficiency, regurgitation, dissection, or aneurysm; recent illness or heart catheterizations within the past 3 months; acute coronary syndrome; diagnosed glaucoma; and pregnancy		Insulin dependence for glycemic control, any major illness in the prior 3 months, previous treatment with EECP therapy, participation in moderate-intensity exercise for 20 min, 2 or more times per week, history of deep vein thrombosis, phlebitis, stasis ulcer and (or) pulmonary embolism, aged less than 21 years or greater than 75 years, uncontrolled hypertension (defined as a systolic blood pressure of 180 mm Hg or more and (or) a diastolic blood pressure of 110 mm Hg or more, measured as the average of at least two readings, obtained at different occasions), systemic hypotension, cardiac arrhythmia that would significantly interfere with the triggering of the EECP therapy device and acute coronary syndrome, such as unstable angina or acute myocardial infarction	
EECP therapy treatment schedule	21 sessions, including one familiarization session, over a seven-week period. Sessions were scheduled with at least 1 day between treatments, aiming to achieve 3 sessions/ week		35 one-hour daily sessions of EECP therapy for 7 consecutive weeks with target inflation pressures of 300 mm Hg		35 one-hour sessions of EECP for 1 h daily 5 days a week for 7 consecutive weeks with target inflation pressures of 300 mm Hg	

*All values have been expressed as mean ± standard deviation (SD)*

*BMI = body mass index; DBP = diastolic blood pressure; ECG = electrocardiogram; EECP = enhanced external counter-pulsation; FPG = fasting plasma glucose; h = hour; HbA1c = glycated haemoglobin; NYHA = New York Heart Association; SBP = systolic blood pressure; SD = standard deviation.*

The key findings of the study and the side effect profile of EECP therapy are summarized in *Table 2*. Funnel plots were plotted to evaluate publication bias and are shown in Supplementary Figure S1.

## Discussion

Standard EECP therapy consists of around 35 1-hour sessions, which typically occur once per day from Monday to Friday.^2^ A maximum of two sessions per day can take place, subject to the patient's wishes and tolerance. Nearly a fifth of the patients who are not able to complete the 35-sessions program may need extended therapy.^2,18^ Commonly accepted contraindications for EECP therapy include arrhythmias that interfere with machine triggering, bleeding diathesis, active thrombophlebitis, severe peripheral artery disease, severe aortic valve disease, prior history of aortic surgery and severe tachycardia (>120 beats/min).^18^ Decompensated homeostasis needs to be stabilized before considering EECP therapy.

Our analysis noted an impressive reduction of -0.70% in HbA1c level in participants with T2DM or prediabetes following EECP therapy completion. These patients had EECP therapy for glycaemic control and had no major underlying cardiac disease. Notably, this reduction in HbA1c is not transient but last even up to 7–12 weeks after completing therapy. HbA1c level reduced by 1.04% and 0.98% at 2–4 weeks and 7–12 weeks after completing therapy, respectively. However, the glycaemic durability of EECP therapy for glycaemic control beyond 12 weeks is not known. Hence, longer follow-up studies investigating glycaemic durability are warranted for determining whether repeat EECP therapy follow-up sessions are needed in patients with T2DM.

**Table 2:** Summary of findings of the key outcomes of this meta-analysis comparing external counter pulsation with placebo in type 2 diabetes mellitus

Outcomes	Anticipated absolute effects^*^ (95% CI)		Relative effect (95% CI)	N° of participants	Certainty of evidence (GRADE)
	Risk with placebo	Risk with EECP			
HbA1c	Mean: 7.75%	MD: 0.7 lower (0.95 lower to 0.45 lower)	-	53 (2 RCTs)	⨁⨁⨁⨁ High
FPG	Mean: 9.05 mmol/L	MD: 1.17 lower (1.56 lower to 0.77 lower)	-	53 (2 RCTs)	⨁⨁⨁⨁ High
2-h PPG	Mean: 15.8 mmol/L	MD: 2.35 lower (3.49 lower to 1.21 lower)	-	48 (2 RCTs)	⨁⨁⨁◯ Moderate^a^
IL-6	Mean: 2.1 pg/ml	MD: 0.5 lower (1.16 lower to 0.17 higher)	-	53 (2 RCTs)	⨁⨁◯◯ Low^b^
Adverse events	0 per 1,000	0 per 1,000 (0 to 0)	OR 2.36 (0.09 to 68.60)	71 (3 RCTs)	⨁⨁⨁⨁ High
ECP therapy compared with placebo at 2–4 weeks for T2DM					
HbA1C	Mean: 7.70%	MD: 1.04 lower (1.32 lower to 0.77 lower)	-	53 (2 RCTs)	⨁⨁⨁⨁ High
ECCP therapy compared with placebo at 7–12 weeks for T2DM					
HbA1C	Mean: 7.65%	MD 0.98 lower (1.22 lower to 0.74 lower)	-	53 (2 RCTs)	⨁⨁⨁⨁ High

*The risk in the intervention group (and its 95% confidence interval) is based on the assumed risk in the comparison group and the relative effect of the intervention (and its 95% CI).

*a. Considerable heterogeneity present as I^2^=78%.*

*b. Considerable heterogenity present as I^2^=85%.*

*CI = confidence interval; EECP = enhanced external counter-pulsation therapy; FPG = fasting plasma glucose; GRADE = Grades of Recommendation, Assessment, Development and Evaluation; h = hour; HbA1c = glycated haemoglobin ; IL-6 = interleukin 6; MD = mean difference; OR = odds ratio; PPG = post-prandial glucose ; PPG = post-prandial glucose ; RCT = randomized controlled trials; T2DM = type 2 diabetes mellitus.*

A similar reduction was also noted in FPG and 2-hour PPG. It is also important to note that a significant reduction in blood pressure was also noted with EECP therapy at the end of the therapy, which persisted till about 2–4 weeks after therapy and thereafter became not significant after 7–12 weeks of therapy. EECP therapy was well tolerated without any major side effects warranting treatment discontinuation.

Our analysis shows that EECP therapy is effective in reducing blood glucose and blood pressure in people with diabetes without CAD. This reduction in blood glucose was accompanied by a reduction in hs-CRP levels, which is a measure of systematic inflammation. However, interleukin-6 levels were similar in the study and the control groups. Our analysis is limited by the small number of patients evaluated. Hence, we need bigger trials with a larger number of people with T2DM with a longer duration of follow-up. The rationale for analysing both people with T2DM and prediabetes together, as was done in one of the RCTs, is that dysglycaemia is a continuum, and there is no reason for EECP therapy not to work in prediabetes if it works in T2DM.^17^ Furthermore, the number of patients was too small to analyse data from people with prediabetes to be analysed separately and is a limitation of this meta-analysis.

Our analysis supports the use of EECP therapy as an adjunctive therapy in people on polypharmacy for T2DM. EECP therapy may help to lower the pill burden or reduce the total daily dose of insulin requirement in these patients. However, these data need to be confirmed by bigger, multicentre clinical studies before they can be replicated in clinical practice. Furthermore, the process of EECP therapy needs to be simplified to be implemented on a large scale; as of today, this is not possible. The cost also remains a major barrier to routinely using EECP therapy, which is a required treatment for a chronic condition such as T2DM. Furthermore, patients with T2DM must go to a medical centre to undergo EECP therapy; this represents a major limitation to using EECP for glycaemic control. Finally, EECP therapy is a time-consuming procedure; consequently, it interferes with the personal and professional lives of the patients.

An important corollary that can be derived from our analysis is that people with T2DM on EECP therapy for angina due to CAD need to reduce the dose of their antidiabetes medication (oral antidiabetes medications and/or insulin) to prevent the risks of hypoglycaemia during and up until at least 12 weeks after EECP therapy completion. Similarly, these patients would also need to modulate the doses of their hypertension medications.

The current systematic review and meta-analysis have a few limitations. First, data are available only up to 12 weeks after EECP therapy completion. What happens beyond that point is not known. The total duration of glycaemic durability of EECP therapy remains to be determined, and it represents an important area of future research in EECP therapy. Second, our analysis included data from only 3 RCTs because not enough RCTs have been published on this subject. Hence, RCTs are urgently needed to evaluate the different metabolic aspects of EECP therapy. EECP therapy has been shown to improve post-exercise recovery in elite rugby league players.^19^ It has also been shown to be beneficial in CAD, one of the macrovascular complications of diabetes, thus improving the quality of life in these patients.^20^ EECP therapy improves exercise tolerance in people with CAD.^21^ EECP therapy has also been shown to reduce the risk of contrast-induced nephropathy in patients with T2DM with CAD undergoing coronary angiography or percutaneous coronary intervention.^22^ A few small studies have suggested the beneficial impact of EECP therapy on transcranial Doppler middle cerebral artery flow velocities and National Institutes of Health Stroke Scale scores in patients with acute ischaemic stroke.^23,24^ The impact of EECP therapy on other macrovascular and microvascular complications of diabetes is not known and remains an important area of future research.

To conclude, EECP therapy is effective in reducing blood glucose and blood pressure in people with T2DM, whose effects extend till at least 7–12 weeks of therapy completion. Hence, blood glucose and blood pressure should be routinely monitored in patients with angina undergoing EECP therapy with suitable modulation of drug doses to prevent hypoglycaemia and hypotension.
